# EZH2-Mediated H3K27me3 Targets Transcriptional Circuits of Neuronal Differentiation

**DOI:** 10.3389/fnins.2022.814144

**Published:** 2022-05-12

**Authors:** Serena Buontempo, Pasquale Laise, James M. Hughes, Sebastiano Trattaro, Vivek Das, Chantal Rencurel, Giuseppe Testa

**Affiliations:** ^1^Department of Experimental Oncology, European Institute of Oncology IRCCS, Milan, Italy; ^2^Department of Oncology and Hemato-Oncology, University of Milan, Milan, Italy; ^3^Human Technopole, Milan, Italy; ^4^Department of Structural Biology and Biophysics, Biozentrum of the University of Basel, Basel, Switzerland

**Keywords:** EZH2 (enhancer of zeste homolog 2), H3K27me3 – histone H3 tri-methylated at Lysine 27, neuronal differentiation, PRC2 (Polycomb repressive complex 2), corticogenesis, epigenetics, transcriptomics, *Prdm13*

## Abstract

The Polycomb Repressive Complex 2 (PRC2) plays important roles in the epigenetic regulation of cellular development and differentiation through H3K27me3-dependent transcriptional repression. Aberrant PRC2 activity has been associated with cancer and neurodevelopmental disorders, particularly with respect to the malfunction of sits catalytic subunit EZH2. Here, we investigated the role of the EZH2-mediated H3K27me3 apposition in neuronal differentiation. We made use of a transgenic mouse model harboring *Ezh2* conditional KO alleles to derive embryonic stem cells and differentiate them into glutamatergic neurons. Time course transcriptomics and epigenomic analyses of H3K27me3 in absence of EZH2 revealed a significant dysregulation of molecular networks affecting the glutamatergic differentiation trajectory that resulted in: (i) the deregulation of transcriptional circuitries related to neuronal differentiation and synaptic plasticity, in particular LTD, as a direct effect of EZH2 loss and (ii) the appearance of a GABAergic gene expression signature during glutamatergic neuron differentiation. These results expand the knowledge about the molecular pathways targeted by Polycomb during glutamatergic neuron differentiation.

## Introduction

The cell-autonomous programs of neuronal differentiation are quintessentially regulated by epigenetic mechanisms determining the ability of a common progenitor to switch fate over time. Indeed, glutamatergic neuron specification in the cerebral cortex is a fine-tuned process that requires accurate timing and spatial organization synchronized by multiple epigenetic players, primarily Polycomb Group Proteins (PcGs) (Yoon et al., 2018). PcGs were first discovered in drosophila as pivotal in regulating the transcription of Hox genes, whose altered expression leads to impaired body segmentation (Kyba and Brock, 1998). Polycomb is constituted by two main repressive complexes involved in cell differentiation during development, PRC1 and PRC2. The PRC2 complex is constituted by four main factors (EED, SUZ12, EZH1/2, and RBAP46/48) and additional proteins not required for enzymatic activity, but necessary to guarantee the optimal efficiency (Margueron and Reinberg, 2011). Its main catalytic subunit, Enhancer of Zeste 2 (EZH2), is responsible for the methylation of lysine 27 on histone H3 (H3K27me1/2/3), a well-known marker associated to transcriptional repression (Cao et al., 2002). EZH2 has been extensively studied during the reprogramming of mouse embryonic fibroblasts into induced pluripotent stem cells (iPSCs), where, despite its inactivation, H3K27me3 is deposited on a selected group of PRC2 targets enriched in developmental regulators that mediate the expression of lineage specific genes (Fragola et al., 2013). Indeed, several groups demonstrated that while PRC2 is dispensable for embryonic stem cells (ESCs) maintenance, it is instead essential to maintain their plasticity during embryonic development by repressing developmental regulators (O’Carroll et al., 2001; Pasini et al., 2007; Chamberlain et al., 2008). Importantly, deletion of *Ezh2* in ESCs results in loss of neurogenic capacity (Pasini et al., 2007).

At the beginning of cortical neurogenesis, neural progenitors called radial glial stem cells undergo asymmetric divisions generating neurons, either directly or through the production of basal progenitors that leave the apical surface of the ventricular zone and move into the subventricular zone (SVZ). The six layers of the cortex are then generated in an inside-out pattern with deep layers produced first and upper layers produced later (Kriegstein and Alvarez-Buylla, 2009; Martynoga et al., 2012). It is known that different sets of genes are differentially regulated by Polycomb during neurodevelopment, resulting in the fine-tuned control of the timing of neural maturation. In particular, only a subset of PRC2 targets are specified in ESCs and, upon cell fate commitment, novel lineage specific genes become transiently H3K27 trimethylated in progenitor cells, including genes that are important for subsequent steps of neuronal maturation (Mohn et al., 2008). The active repression of non-neuronal genes mediated by PRC2 in adult neurons is needed for bivalent genes (marked by both H3K27me3 and H3K4me3) and this repression may change due to intrinsic or extrinsic cell stressors (Burgold et al., 2012; Corley and Kroll, 2015). During cortical neuron differentiation, EZH2 down-regulation and EZH1 up-regulation occur in a time-regulated fashion, directing not only the timing of neuronal maturation but also the neurogenic to astrogenic switch (Testa, 2011).

The evidence that EZH2 acts as a molecular switch during cortical development is corroborated by different studies that addressed part of the EZH2-mediated regulation by abolishing its function in different phases of corticogenesis. *Ezh2* knockdown (KD) in the neural tube of chicken embryos induces defects in the apico-basal polarity of neuroblasts causing impaired neural tube organization, which is mediated by the cell cycle regulator p21Waf1/Cip1 (Akizu et al., 2016). Instead, loss of function of *Ezh2* in mouse cortical progenitor cells before the onset of neurogenesis changes the balance between differentiation and self-renewal toward differentiation (Pereira et al., 2010). Indeed, despite a broadly conserved temporal order of differentiation, the neurogenic period was found to be shorter in absence of EZH2, with a reduced neuronal output and unbalanced representation of deep and upper layer neurons toward the former. Conversely, ablation of *Ezh2* at the neurogenic to astrogenic switch period leads to the late onset of astrogenesis due to a prolonged neurogenic phase, in turn caused by the increase of Ngn1 mRNA levels, a direct PRC2 target (Hirabayashi et al., 2009).

In addition to the molecular evidence that EZH2 is pivotal for regulating corticogenesis in mice, haploinsufficiency of *EZH2* in humans causes intellectual disability in the context of Weaver Syndrome (WS), a rare disease characterized by overgrowth, facial dysmorphism, and intellectual disability (Weaver et al., 1974). In 2011, WS was found to be caused by *de novo* mutations in PRC2 members *EZH2* and *EED* (Tatton-Brown et al., 2011; Gibson et al., 2012; Cohen et al., 2015). The *EZH2* mutations identified in WS patients occur heterozygously, mostly in conserved residues of the catalytic SET domain, resulting in *EZH2* haploinsufficiency due to loss of function of the mutated allele (Lui et al., 2018).

Even though the role of EZH2 during corticogenesis has started to be elucidated, our knowledge is limited to a few targets and to a general understanding of the PRC2 function in the regulation of the timing of neuronal differentiation, though without a deep comprehension of the dysregulation propagated in neurons during their fate specification. In this work, we investigate the role of EZH2 in regulating gene transcription during glutamatergic neuronal differentiation, with a focus on mature post-mitotic neurons. By combining ChIP-seq and RNA-seq experiments we report that *Ezh2* ablation leads to aberrant up-regulation of genes related to neuronal maturation, and particularly to synaptic plasticity, as well as novel epigenetic mechanisms possibly linking EZH2 to the regulation of the neuronal fate specification.

## Results

### Establishment of an *Ezh2* Conditional Deletion Model to Study Its Role During Terminal Neuronal Differentiation

**FIGURE 1:**
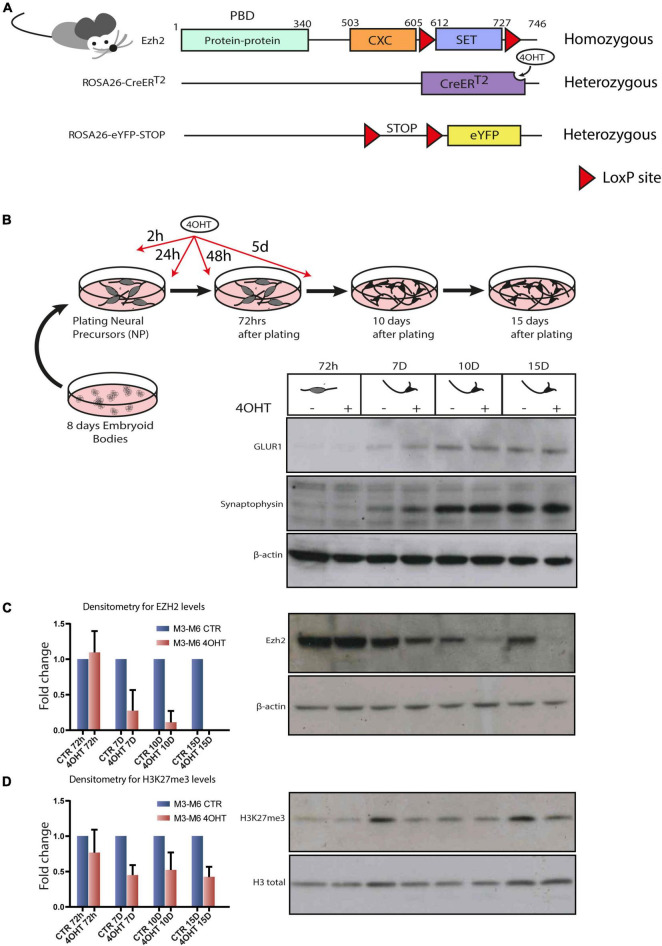
Establishment of an Ezh2 conditional deletion model to study its role during terminal neuronal differentiation. **(A)** Schematic of transgenic mouse strain Ezh2*^flox/flox^* rosa26 (CreERT2)/rosa26-eYFP used for the conditional deletion of Ezh2. **(B)** Graphical representation of the neuronal differentiation protocol used to derive mature post-mitotic glutamatergic neurons. Western blots for neuronal markers GLUR1 and Synaptophysin at 72h, 7D, 10D and 15D after neuronal progenitors plating using β-actin as loading control. **(C)** Representative western blot for EZH2 and densitometric analysis expressed as fold change of 4OHT samples compared to controls for every time point. The analysis was performed on 2 independent biological replicates from independent cell lines carrying the Ezh2*^flox/flox^* allele (M3 and M6) (mean ± SD, one sample *t*-test). **(D)** Representative western blot for H3K27me3 and densitometric analysis relative to histone H3 total signal. The analysis was performed on 2 independent biological replicates from independent ESC lines carrying the Ezh2*^flox/flox^* allele (M3 and M6) (mean ± SD, one sample *t*-test).

In order to study the specific effect of disabling EZH2 enzymatic activity in post-mitotic neurons, we set to derive murine embryonic stem cell lines (mESC) harboring the following alleles: (i) the floxed *Ezh2* SET domain in homozygosis, (ii) the inducible Cre-recombinase in the *ROSA26* locus in heterozygosis, and (iii) the eYFP, as a control of Cre activation in the *ROSA26* locus in heterozygosis. To this end, we crossed homozygous mice for the floxed SET domain of *Ezh2* (Su et al., 2003) with mice harboring a 4-hydroxytamoxifen (4-OHT) inducible Cre-recombinase (CreER^*T*2^) into the *ROSA26* locus (Seibler et al., 2003) (hereafter *Ezh2*^flox/flox^*/CreERT2^/+^*). We crossed animals from this newly generated line with others carrying *eYFP* into the *ROSA26* locus, of which expression was possible after removal of a floxed STOP cassette (hereafter *Ezh2*^flox/flox^*/CreERT2^/+^/eYFP^/+^*). The final genomic organization of the engineered *loci* is depicted in Figure 1A. The presence of both the *Ezh2* and *ROSA26* loci on chromosome 6 generated the possibility of intra-chromosomic recombination, because of the presence of two, although very distant, floxed cassettes. We excluded this possibility by performing copy number assay for *Cnbp*, a gene located between the two cassettes (Supplementary Figure 1A). This particular chromosomal configuration impinged on the derivation of different genotypes, amongst which the *Ezh2*^WT/WT^*/CreERT2^/+^/eYFP^/+^* one. However, we were able to derive blastocysts from *Ezh2*^flox/flox^*/CreERT2^/+^/eYFP^/+^* transgenic mice and to established two embryonic stem cell (ESCs) lines referred to as M3 and M6. We validated the Cre-mediated recombination by Western blot analysis (Supplementary Figure 1B).

Given our interest in studying the consequences of EZH2 impairment in the cortex, we differentiated the *Ezh2*^flox/flox^*/CreERT2^/+^/eYFP^/+^* mESCs lines following the protocol outlined in Figure 1B that, upon neuronal progenitors differentiation in embryoid bodies and their subsequent plating in 2D, yields a 95% pure population of glutamatergic neurons in 15 days (Bibel et al., 2004). We treated neural progenitors with 4-OHT at a concentration of 300 nM in four pulses at 2h, 24h, 48h and 5d after plating of neuronal progenitors. We then confirmed the quality of neural differentiation achieved by the appearance of neuronal markers such as Synaptophysin and GLUR1 (Figure 1B) and the decrease of the apical progenitors’ marker PAX6 and of the proliferation marker Ki67 after 72 h (Supplementary Figure 1D). The full-length *Ezh2* mRNA levels were markedly reduced at 72 h (Supplementary Figure 1C). The protein, which showed a high stability in this cellular type, was drastically reduced after 7-10 days (7D-10D) and even further at 15 days (15D), as confirmed by densitometric analysis (Figure 1C). This model allowed us to study specific effects of EZH2 loss of function on executing the neuronal differentiation program starting from neuronal progenitors.

### EZH2 Impairment Causes Global Loss of the H3K27me3 Mark in Differentiating Neurons

We focused our attention at 72 h, when EZH2 protein levels in treated and untreated cells were still similar, and at 10D and 15D after plating of neuronal progenitors, when the levels of EZH2 in treated cells were strongly reduced. Western blot and densitometric analysis for H3K27me3 revealed a reduction of this mark upon EZH2 deletion (Figure 1D).

**FIGURE 2:**
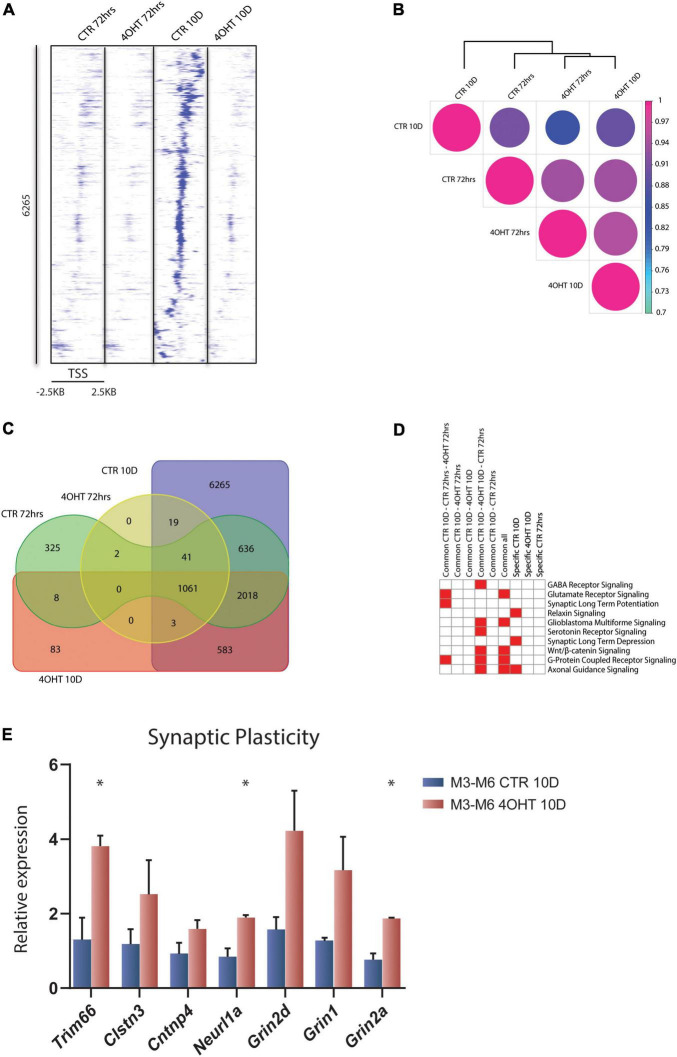
EZH2 impairment causes global loss of the H3K27me3 mark in differentiating neurons. **(A)** Heat map of normalized tag densities, representing the CTR10D-specific H3K27me3 marked peaks around promoters. Each row represents a 5-kb window centered on the gene TSS and extending 2.5 kb upstream and 2.5 kb downstream. The signal has been generated by merging the bam files of the biological replicates. **(B)** Correlation matrix plot and hierarchical clustering of the 4-OHT and CTR samples based on H3K27me3 distribution around the TSS. **(C)** Venn diagram showing the overlap of H3K27me3 enriched peaks around promoters (± 2.5kb TSS) between 4-OHT and CTR samples at 72 h and 10D. **(D)** Heat map showing the top 10 significantly enriched canonical pathways (*p*-value < 0.001) related to neuronal function. *P*-values were computed using IPA software. **(E)** qRT-PCR validation of selected genes involved in the LTD and NMDA receptor pathways. The experiment was performed on 2 independent neuronal differentiations from 2 independent ESC lines (M3 and M6) (mean ± SEM, unpaired *t*-test) (*: *p* value < 0.05).

To investigate the dysregulation caused by *Ezh2* deletion during neural differentiation, we interrogated the genome-wide distribution of H3K27me3 using ChIP-seq. We analyzed the time points of 72 h and 10D after plating of neuronal progenitors. In this temporal window, the establishment of the EZH2 KO happened starting from a condition of similarity between controls (CTR) and treated (4-OHT) samples at 72 h to a condition of marked reduction EZH2 in 4-OHT samples at the 10D time-point (Figure 1C). Direct comparison of the H3K27me3 targets across all the samples highlighted an impairment of the acquisition of H3K27me3 in 4-OHT10D, with 6,265 peaks around promoters (± 2.5kb around the TSS), corresponding to 3,456 genes (Supplementary Table 1), that specifically acquired this histone mark in presence of EZH2, but not upon its KO (Figure 2A). Unsupervised cluster analysis of H3K27me3 profiles across CTR and 4-OHT samples showed that CTR10D clustered apart from CTR72h and 4-OHT samples (Figure 2B), confirming that 4-OHT10D neurons largely failed the acquisition of H3K27me3 at promoters. We then looked at the overlap of H3K27me3 enriched peaks between 4-OHT and CTR samples at 72 h and 10D (Figure 2C). Canonical pathways analysis on each set of genes found in Figure 2C unveiled enrichment for pathways involved in neuronal development and maturation (Figure 2D). A complete list of the enriched categories is depicted in Supplementary Figure 2. Importantly, synaptic long-term depression (LTD) was selectively enriched in CTR10D (Figure 2D), suggesting that genes involved in this pathway were repressed in CTR, while erroneously upregulated upon EZH2 impairment. We confirmed this hypothesis by qRT-PCR on 2 independent cell lines for both genes involved in LTD (Trim66, Clstn3, Cntnp4 and Neurl1a) and members of the NMDA receptor (Grin1, Grin2a, Grin2d), known to play an important role in synaptic plasticity (Gaiarsa et al., 2002; Figure 2E).

Our data showed loss of H3K27me3 upon EZH2 impairment at a subset of promoters and revealed that the expression of genes involved in synaptic plasticity, and particularly to LTD, was dysregulated in 4-OHT differentiating neurons.

### EZH2-Dependent Transcriptional Modulation Converges With PRDM13 Regulatory Network

**FIGURE 3:**
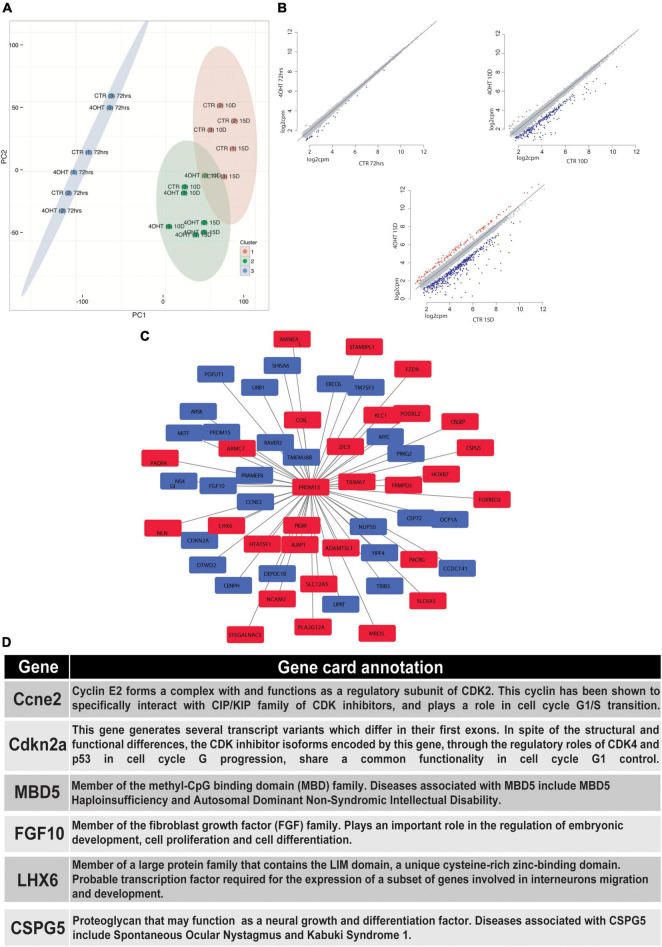
EZH2-dependent transcriptional modulation converges with PRDM13 regulatory network. **(A)** Principal component analysis of 4-OHT and CTR samples at 72h, 10D and 15D after plating of neuronal progenitors based on gene expression. **(B)** Scatter plots showing differentially upregulated (red dots) and downregulated (blue dots) genes with fold change > 2 and FDR < 0.05 in 4-OHT samples at 72h, 10D and 15D after plating of neuronal progenitors; axes values correspond to log2cpm (counts per million reads) in CTR samples (x) and 4-OHTsamples (y). **(C)** PRDM13 regulatory network computed integrating 10D differential expression data with a co-expression network analysis performed using the GeneMania database (Warde-Farley et al., 2010). Red squares indicate genes with a concordant direction of expression as compared to Prdm13 whereas blue squares indicate genes with a discordant direction of expression as compared to Prdm13 **(D)** Gene card annotation and status in the differential expression analysis in 4-OHT10D samples of 6 relevant genes of the PRDM13 regulatory network depicted in Figure 3C.

In order to correlate the EZH2-dependent H3K27me3 loss with the transcriptional modulation during neural differentiation, we performed RNA-seq of CTR and 4-OHT samples at 72 h, 10D, and 15D after neuronal progenitors plating. Principal component analysis (PCA) based on transcriptomics profiles followed by k-means clustering identified three clusters: (i) the first was constituted by CTR and 4-OHT samples at 72 h; (ii) the second by CTR samples at 10D and 15D; and (iii) the third by 4-OHT samples at 10D and 15D (Figure 3A). These results corroborated the levels of EZH2 and H3K27me3 observed by Western blot at the same time points (Figures 1C,D). We found that one of the three replicates of the CTR10D clustered with the group of the 4-OHT samples, however unsupervised hierarchical clustering of 4-OHT and CTR samples showed that the CTR10D outlier seen by PCA clustered with the other control samples when analyzing the top variant genes (Supplementary Figure 3).

Differential expression analysis between CTR and 4-OHT samples at each time point revealed, as expected, small differences at 72 h with only 30 differentially expressed genes (DEGs) with FDR < 0.05 and FC > 2 (Supplementary Table 2). The same analysis at advanced time points showed significant changes in transcriptional programs. Interestingly, with the same threshold of FDR and FC we found 296 DEGs comparing 4-OHT and CTR samples at 10D of which, surprisingly, only 4% (*n* = 11) were up-regulated (Figure 3B and Supplementary Table 2).

Given the loss of repressive activity on gene expression exerted by EZH2, we hypothesized an alternative mechanism where the up-regulation of a transcriptional repressor could promote the down-regulation of most of the DEGs, partially explaining our results. Among the top up-regulated genes in the 4-OHT10D samples we found *Prdm13*, a transcriptional repressor well known for its role in mediating the balance between inhibitory and excitatory neurons, and in defining neuronal identity (Mona et al., 2017).

We then investigated the transcriptional programs related to PRDM13 expression by reconstructing a PRDM13 regulatory network through the integration of our context-specific differential expression data with a co-expression network analysis performed using the GeneMania database (Warde-Farley et al., 2010; Figure 3C). In particular, we used the GeneMania networks to identify genes reported to be co-regulated with PRDM13. Interestingly, among *Prdm13* co-expressed genes we found genes involved in the regulation of cell cycle (e.g., *Ccne2, Cdkn2a*) (Masui et al., 2008; Lacomme et al., 2012; Price et al., 2014), genes involved in neuronal differentiation (e.g., *Mbd5, Fgf10*) (Sahara and O’Leary, 2009; Walz and Young, 2014) and genes involved in GABAergic neurons specification and migration (e.g., *Lhx6, Cspg5*) (Liodis et al., 2007; Jüttner et al., 2013; Figures 3C,D).

### Dissection of the Transcriptional Impact of Ezh2 Impairment in the Regulation of Neuronal Gene Expression Programs

**FIGURE 4:**
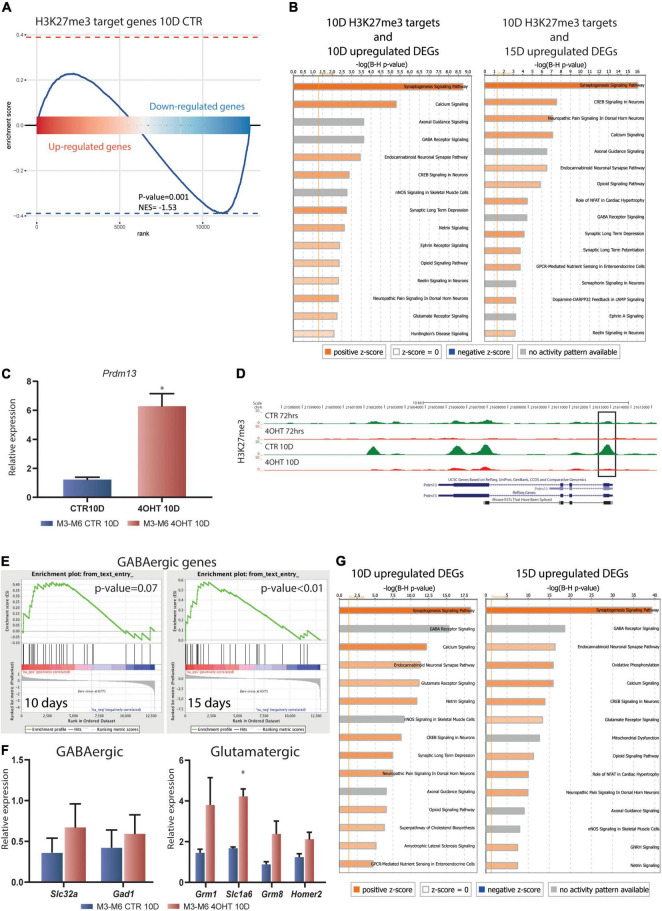
Dissection of the transcriptional impact of Ezh2 deletion unveiled direct and indirect regulation of neuronal gene expression programs. **(A)** Enrichment plot showing the distribution of H3K27me3 targets in the ranked gene expression signature between treated and control samples at 10D. The blue curve represents the enrichment score, the bar in the middle represents the ranked gene expression signature between treated and control samples at 10D. Red and blue indicate up-regulation and down-regulation in the 4-OHT samples, respectively. The *P*-value (0.001) and normalized enrichment score (NES, –1.53) were computed using the fgsea algorithm (Litwin, 1976). **(B)** Canonical pathway analysis performed on the upregulated DEGs (FDR < 0.05) at 10D and 15D (left and right, respectively) found to be H3K27me3 targets in CTR10D. The top 15 pathways are shown. B-H multiple testing corrected p-values were computed using IPA software. **(C)** qRT-PCR validation of Prdm13 expression levels in 10D neurons. The experiment was performed on 2 independent biological replicates from 2 independent ESC lines carrying the Ezh2*^flox/flox^* allele (M3 and M6) (mean ± SEM, unpaired *t*-test) (*: *p* value < 0.05). **(D)** H3K27me3 ChIP-seq tracks for the Prdm13 gene. Black rectangle highlights the peak on the TSS. **(E)** Gene set enrichment analysis showing the enrichment for GABAergic genes in the 4-OHT samples at 10D and 15D. **(F)** qRT-PCR validation of selected genes involved in the GABAergic and Glutamatergic pathways. The experiment was performed on 2 independent biological replicates from 2 independent ESC lines carrying the Ezh2*^flox/flox^* allele (M3 and M6) (mean ± SEM, unpaired *t*-test) (*: *p* value < 0.05). **(G)** Canonical pathway analysis performed on upregulated DEGs (FDR < 0.05) at 10D and 15D (left and right, respectively). The top 15 pathways are shown. B-H multiple testing corrected *p*-values were computed using IPA software.

We assessed the transcriptional impact of the *Ezh2* deletion on the H3K23me3 targets at 10D by integrating ChIP-seq and RNA-seq results. GSEA showed that the transcriptional reprogramming mediated by *Ezh2* deletion significantly affected the H3K27me3 targets in CTR samples. In particular, we found a clear bipartition of H3K27me3 targets that became either up- or down- regulated upon *Ezh2* impairment, with a significant enrichment in the downregulated genes (*p*-value = 0.001, Figure 4A).

We then performed an enrichment analysis for canonical pathways on the genes that specifically preserved H3K27me3 in CTR10D as compared to 4-OHT10D samples and that became upregulated at 10D or 15D upon *Ezh2* deletion (375 and 614 genes, respectively, FDR < 0.05 without threshold on FC). These analyses uncovered that genes involved in synaptogenesis, axonal maturation, and in general neuronal functions are direct EZH2 targets and that its impairment causes their aberrant up-regulation (Figure 4B and Supplementary Tables 3, 4). Interestingly, glutamate receptor signaling and LTD were significantly enriched in this analysis as a further confirmation of an EZH2-dependent dysregulation of these pathways in 4-OHT samples (Figure 4B and Supplementary Tables 3, 4). The same analyses on downregulated EZH2 targets did not show enrichment neuronal-related categories (Supplementary Figures 5A,B and Supplementary Tables 5, 6).

The observation that the *Prdm13* regulatory network might indirectly explain the strong down-regulation observed in RNA-seq led us to the validation of *Prmd13* mRNA levels by qRT-PCR (Figure 4C). As it turns out, we found *Prdm13* up-regulated in two independent cell lines. To prove the relation between EZH2 impairment and *Prmd13* up-regulation, we looked at the H3K27me3 peak at *Prmd13* TSS in our ChIP-seq data, confirming the loss of this histone mark in 4-OHT10D samples (Figure 4D).

Given the demonstrated role of PRDM13 in maintaining the balance of glutamatergic and GABAergic neurons in the cortex, we hypothesized the impairment of this mechanism upon *Ezh2* deletion in our differentiation paradigm, which was reported to produce a 95% pure population of glutamatergic neurons (Bibel et al., 2007). Using gene set enrichment analysis (GSEA) probing known genetic markers for both glutamatergic and GABAergic neurons (Sugino et al., 2006), we found enrichment toward the GABAergic lineage signature at 15D (Figure 4E), and a non-significant enrichment in the glutamatergic lineage signature after 10D and 15D of *Ezh2* deletion (Supplementary Figure 4). We then measured the expression levels of two key GABAergic markers, *Slc32a* (salute carrier family 32, a the vesicular transporter of GABA) and *Gad1* (encoding the glutamate acid decarboxylase enzyme), and several glutamatergic markers *Grim1, Slc1a6, Grim8*, and *Homer2* by qRT-PCR at 10D, the time point in which we observed strong up-regulation of *Prdm13*, finding up-regulation of all of them in two independent differentiations from two different ESC lines (Figure 4F).

Finally, canonical pathway analysis of the upregulated DEGs in 4-OHT samples at 10D and 15D showed enrichment for categories related to neuronal function and differentiation, suggesting an hypothesis of accelerated maturation of EZH2-deficient neurons (Figure 4G and Supplementary Tables 7, 8). Also in this case, canonical pathway analysis for the downregulated DEGs in 4-OHT samples did not show enrichment for neuronal-related categories (Supplementary Figures 5C,D and Supplementary Tables 9, 10).

Together, the integration of H3K27me3 ChIP-seq data and RNA-seq profiles supported that the effect of *Ezh2* deletion during differentiation of glutamatergic neurons unfolds in: i) the dysregulation of genes involved in neuronal differentiation and function, synaptic long term depression, and glutamatergic receptor signaling, with key H3K27me3 targets involved in these pathways up-regulated; and ii) the up-regulation of a GABAergic transcriptional footprint, possibly linked to the up-regulation of *Prdm13* upon EZH2 loss.

## Discussion

By means of conditional inactivation, we were able to study the EZH2-dependent transcriptional programs specific to mESC-derived cortical glutamatergic neurons. We showed that EZH2 is essential for maintaining H3K27me3 in post-mitotic neurons and that the loss of this histone mark, and prevention of its later acquisition, causes a cascade of transcriptional dysregulation. As a result, the expression of multiple genes was affected, including genes related to neuronal maturation and plasticity as well as to cell fate.

The presence of both *Ezh2* and *ROSA26* loci on chromosome 6 made the derivation of conditional *Ezh2* mESCs lines particularly challenging because of the possibility of intra-chromosomic recombination between the two floxed cassettes. Despite extensive rounds of breeding, we obtained low frequency of all desired genotypes, including the *Ezh2*^WT/WT^*/CreERT2^/+^/eYFP^/+^* control that would have been particularly useful to discern Cre-specific effects from the EZH2-dependent ones. The Cre/loxP system has been shown to particularly affect proliferating cells, where it causes genome toxicity (Loonstra et al., 2001; Bohin et al., 2018), and Cre-mediated toxicity in the brain was linked to deleterious effects in neuronal progenitors leading to severe microcephaly (Forni et al., 2006). Moreover, tamoxifen administration alone during early cortical development was reported to affect neuronal progenitors differentiation, in turn leading to several neuronal deficits (Lee et al., 2020). While we obtained only one such control line, which proved however not proficient in differentiation and could thus not be integrated in our design, we ensured that our experimental setup intrinsically averted the issues potentially arising from CreERT2 expression and 4-OHT administration in neuronal progenitors, since 4-OHT treatment started only with the induction of neuronal progenitors’ terminal differentiation. Indeed, 24h after plating of neuronal progenitors, which coincided with the 24 h time point of Cre expression, we observed reduction of progenitors’ markers in both CTR and 4-OHT samples (Supplementary Figure 1D). Importantly, other studies demonstrated that the expression of Cre in adult mice neurons did not cause any toxic effect (Ahmed et al., 2004).

Moreover, the loss of H3K27me3 detected upon EZH2 KO is an indication of specificity of the effects observed. Indeed, after 72 h from neuronal progenitor plating, following 3 pulses of 4-OHT, H3K27me3 levels resembled the EZH2 protein ones (Figures 1C,D), with low reduction of this histone mark and no reduction of EZH2 levels. Only when EZH2 protein was drastically reduced we started to detect bigger H3K27me3 reduction, which persisted till the end of differentiation. This is further corroborated by our RNA-seq results, where 10D and 15D samples clustered according to the presence of EZH2 KO, while 72 h samples clustered apart from the rest and were not separated by treatment, despite 72 h of 4-OHT exposure (Supplementary Figure 3). In addition, differential expression analysis between CTR and 4-OHT at the three stages showed strong dysregulation in 4-OHT samples at 10D and 15D, while the number of DEGs was extremely low at 72 h. Together, these data indicate that the phenotypes reported are specific for EZH2 KO regardless of 4-OHT exposure or CreER^*T*2^ expression.

We used H3K27me3 ChIP-seq data as a readout of the effects of EZH2 KO. Although spike-ins should be used for quantitative claims using ChIP-seq data (Orlando et al., 2014), we exploited H3K27me3 ChIP-seq tracks in combination with RNA-seq profiles, linking the observed loss of H3K27me3 in EZH2 KO samples to the actual variation in gene expression. When we examined the 3,456 targets of Polycomb that failed to acquire the tri-methylation of H3K27 after *Ezh2* deletion, we found a significant enrichment for genes involved in LTD by canonical pathway analysis. This form of synaptic plasticity is an action-dependent adaptation in which neurons become “de-sensitized” to excessive stimuli, such as high levels of (extracellular) Ca^2+^ (Zucker, 1999). One way that neurons can adapt synapse functionality is by changing the subunit composition of ion channel receptors like the NMDA receptor (Yashiro and Philpot, 2008). NMDA receptors are comprised of two obligatory (NR1) and two regulatory (NR2) subunits (Cull-Candy and Leszkiewicz, 2004), have many critical roles in neural plasticity (Ishii et al., 1993), and their malfunctions have been implicated in many neurological disorders (Tarabeux et al., 2011; Lakhan et al., 2013; Lee et al., 2015). In our experiment, we found an up-regulation in the expression of three critical NMDA receptor subunits, Grin1 (NR1), Grin2A, and Grin2D (NR2) (Figure 2E). Interestingly, mutations in Grin2A have been associated with a spectrum of intellectual disorders (Strehlow et al., 2019). The early appearance of NMDA receptor subunits in 4-OHT samples was rather intriguing as it suggested an accelerated neuronal differentiation and misregulation of synaptic plasticity; functional NMDA receptors appear later in neuronal development and are usually potentiated upon stimuli (Monyer et al., 1994; Malenka and Bear, 2004). Importantly, studies performed in an animal model for schizophrenia demonstrated that H3K27me3 deposition controls NMDA receptor expression during postnatal development. In particular, H3K27me3 levels have been found to be increased at *Grin2a* and *Grin2b* promoters, supporting our findings that EZH2 controls NMDAR subunits expression (Gulchina et al., 2017). Our results converge with multiple lines of evidence suggesting a critical role of H3K27me3 in synaptic plasticity. Indeed, conditional KO of the H3K27me3 demethylase UTX causes impairment in long term potentiation (LTP) (Tang et al., 2017). PRC2 is also responsible for the activation of activity-dependent genes upon neuronal stimulation (Palomer et al., 2016), which regulate the late phases of LTP and LTD (Gandolfi et al., 2017). This is particularly true for the neurotrophic growth factor Bdnf, which contains several promoters that present a partially repressed status controlled by PRC2 (Palomer et al., 2016). We demonstrated that an LTD distinctive gene signature loses H3K27me3 at promoters upon *Ezh2* conditional KO in differentiating glutamatergic neurons, a result in line with the reduced amount of this histone mark at Bdnf promoters upon NMDA-mediated induction of LTD (Palomer et al., 2016). Moreover, other studies demonstrated that EZH2 is pivotal for spatial learning, spatial working and recognition memories, and memory reconsolidation through repression of PTEN for controlling the activation of the AKT-mTORC1 (Zhang et al., 2014, 2019; Jarome et al., 2018). Together, our analyses support the increasing body of evidence that EZH2 is essential for the epigenetic control underlying synaptic plasticity and, in particular, LTD, although further experiments are needed to link the transcriptional dysregulation we uncovered to functional alterations.

The differentiation protocol utilized in this work produces a 95% pure population of glutamatergic neurons with less than 1% positive for GABA (Bibel et al., 2007). While deletion of *Ezh2* was shown to accelerate stem cell differentiation to functional neurons (Yu et al., 2011), the final identity and genetic pathways involved are not well studied. Our data suggest that homologous deletion of *Ezh2* at the onset of neuronal progenitor differentiation causes the upregulation of a GABAergic transcriptional signature, which may represent a mechanism through which *Ezh2* regulates neuronal fate in physiological development, although further experiments are needed to confirm this hypothesis.

A possible explanation for this transcriptional phenotype comes from the ChIP-seq analysis, where we found that *Prdm13* lost H3K27me3 and was upregulated upon deletion of *Ezh2*. Our transcriptomics data show, that upon *Ezh2* KO, after 10D of neuronal progenitor differentiation 296 genes were upregulated with FDR < 0.05 and FC > 2, however only 4% of them were strongly up-regulated. This finding resulted a counterintuitive finding considering the transcriptional repressive role of PRC2. Interestingly, among the strongly up-regulated genes with FC > 2, *Prdm13* resulted of particular interest in light of its reported repressive activity during many developmental processes, including neuronal differentiation (Chang et al., 2013; Mona et al., 2017). PRDM13 belongs to the Prdm gene family, characterized by the presence of a conserved PR domain, structurally and functionally related to the SET methyltransferase domain, at the N-terminus of the protein followed by an array of zinc fingers (Buyse et al., 1995; Fumasoni et al., 2007; Kinameri et al., 2008; Sun et al., 2008; Fog et al., 2012; Hohenauer and Moore, 2012). Despite the presence of the PR domain, only a subset of Prdm proteins have intrinsic catalytic activity (Buyse et al., 1995; Derunes et al., 2005; Hayashi et al., 2005; Eom et al., 2009), and, indeed, their function is not limited to the histone methyltransferase activity, but can also occur through the recruitment of different chromatin modifiers and transcriptional repressors at gene promoters in a context-dependent fashion (Fog et al., 2012; Hohenauer and Moore, 2012). PRDM13 function is debated in literature and, although researchers have reported methyltransferase activity with unknown roles *in vivo* (Chang et al., 2013), its PR domain is quite divergent from the classical SET domains (Chang et al., 2013). Conversely, other groups have demonstrated that the PR domain is dispensable for PRDM13 activity, highlighting the pivotal role of the zinc finger array for its function, thus providing evidence for a tethering role of PRDM13 in the recruitment of chromatin remodelers mediating transcriptional repression (Chang et al., 2013).

Here, we demonstrated that the H3K27 trimethylation at *Prdm13* promoter is severely reduced when EZH2 function is impaired in differentiating glutamatergic neurons (Figure 4D). PRDM13 was reported to play a critical role in the specification of neuronal identity by keeping ventral cell-type genes silenced in the dorsal neural tube (Mona et al., 2017). PRDM13 prevents neural bHLH factors from activating transcription by binding the same chromosomic regions. The bHLH transcriptional activator PTF1A is responsible for the activation of *Prdm13* transcription (Nakada et al., 2004; Yu et al., 2011), which in turns inhibits *Ptf1a* transcription in a negative feedback loop, thereby ensuring its expression in a precise temporal window (Nakada et al., 2004). Moreover, PRDM13 is predicted to be recruited by NEUROG1, NEUROG2, and ASCL1 (Chang et al., 2013; Mona et al., 2017), and, indeed, its loss of function results in ectopic expression of multiple bHLH regulated genes in the dorsal neural tube (Mona et al., 2017).

In the mouse spinal cord, PTF1A and ASCL1 act upstream of homeodomain factors such as PAX2 and TLX1/TLX3, important for GABAergic and glutamatergic neuron specification, respectively (Nakada et al., 2004; Cheng et al., 2005; Bröhl et al., 2008; Chang et al., 2013; Borromeo et al., 2014). Interestingly, both *Ascl1* and *Ptf1a* are expressed in GABAergic neuron progenitors (Beres et al., 2006; Masui et al., 2008) and the molecular mechanism through which the glutamatergic fate is repressed was attributed to PRDM13, being sufficient to promote the GABAergic fate by directly repressing *Tlx1* and *Tlx3*, thus indirectly leading to *Pax2* up-regulation (Cheng et al., 2005; Chang et al., 2013). These results are corroborated by experiments in chick neural tubes, where *Prmd13* overexpression induces the GABAergic fate (Chang et al., 2013). Importantly, EZH2 has already been linked to GABAergic interneuron differentiation in the cerebellum through direct control of *Ptf1a* and *Pax2* expression (Feng et al., 2016). Our results show that the up-regulation of *Prdm13* mediated by *Ezh2* loss of function may be responsible for the appearance of a mixed GABAergic and glutamatergic transcriptional signature (Figures 4C-F) when pushing embryonic stem cells toward the glutamatergic fate. Although further experiments are needed to dissect this transcriptional phenotype, we explored the *Prdm13* regulatory network by computational inference, given the difficulties in performing PRDM13 ChIP-seq due to the unavailability of a commercial antibody (Mona et al., 2017). We did so by integrating our context-specific gene expression data and a co-expression network analysis performed using the GeneMANIA database (Warde-Farley et al., 2010). We found that the *Prdm13*-centered regulatory network includes genes involved in neuronal differentiation and genes responsible for cell cycle control (Figures 3C,D). Indeed, Mbd5, up-regulated in our datasets, is responsible for the correct neurite outgrowth and its deletion causes intellectual disability (Walz and Young, 2014). Regulation of Fgf10 expression, found down-regulated in our experiments, is instead crucial for early neural progenitor expansion and onset of neurogenesis (Sahara and O’Leary, 2009). Our analyses also suggest that PRDM13 may mediate cell cycle regulation through *Ccne2* and *Cdkn2a*, two genes we found down-regulated upon *Ezh2* deletion and important for neuronal differentiation (Lacomme et al., 2012; Price et al., 2014; Feng et al., 2016). Importantly, in line with our results suggesting that *Prdm13* overexpression may be responsible for a GABAergic/glutamatergic differentiation imbalance, we also show that PRDM13 regulatory network includes two genes up-regulated upon *Ezh2* KO: i) *Lhx6*, involved in GABAergic neurons specification and migration (Liodis et al., 2007), and ii) *Cspg5*, that if deleted causes impairment in GABAergic synapses maturation (Jüttner et al., 2013).

Interestingly, the experimental setting used for this study reproduced *in vitro* what Pereira and colleagues (Pereira et al., 2010) performed *in vivo*, namely the *Ezh2* KO at the time of neurogenesis onset. Their work demonstrated that *Ezh2* loss of function at the onset of neurogenesis causes accelerated differentiation. The data we report show that the glutamatergic signature is overall not significantly over-represented in *Ezh2* KO neurons (Supplementary Figure 4), but some of the genes that constitute it are up-regulated (Figure 4F), suggesting a possible temporal acceleration of the glutamatergic differentiation not reflected in an up-regulation of the overall glutamatergic signature. As a further evidence of this, canonical pathway analysis of direct EZH2 targets that we found up-regulated upon its impairment at 10D and 15D, as well as of the overall up-regulated DEGs in 4-OHT samples at the same time points, showed an enrichment for categories related to neuronal function and maturation, corroborating the hypothesis of faster neuronal differentiation in absence of functional EZH2 (Figures 4B,G).

Taken together, our findings point at precise dysregulations in neuronal homeostasis upon EZH2 loss that may represent an entry point for the study of the intellectual disability characterizing Weaver Syndrome. As a matter of fact, EZH2 haploinsufficiency is the main cause of this disease (Lui et al., 2018), therefore the understanding of the EZH2 loss of function effects in differentiating neurons adds valuable knowledge to its already established roles during cortical development and paves the way for more translational studies.

## Materials and Methods

### Murine Strains

Experiments involving animals have been performed in accordance with the Italian Laws (D.L.vo 116/92 and following additions), which enforced EU 86/609 Directive (Council Directive 86/609/EEC of 24 November 1986 on the approximation of laws, regulations and administrative provisions of the Member States regarding the protection of animals used for experimental and other scientific purposes). Mice have been housed accordingly to the guidelines set out in Commission Recommendation 2007/526/EC - June 18, 2007 on guidelines for the accommodation and care of animals used for experimental and other scientific purposes. The Italian legislation, at the time of approval of these experiments, did not require a specific ethical review process for all the experiments involving animals. A central (Government) review was required only for particular species (e.g., dogs, cats, and non-human primates) or for experiments done without anesthesia or that will or may cause severe pain. In all other cases, only a notification of the experiments to the Ministry of Health was required. Accordingly, the experiments involving animals included in this paper have been notified to the Italian Ministry of Health in 2009, before ARRIVE guidelines implementation.

*Ezh2^fl^* mice, rosa26 (CreER^*T*2^) mice and rosa26-eYFP have been previously described (Srinivas et al., 2001; Seibler et al., 2003; Su et al., 2003). The triple mutants were generated by intercrossing these three individual strains. Primers used for genotyping are listed in Supplementary Table 11.

### Derivation, Culture and Differentiation of ES Cells

To derive embryonic stem cells, plugged female mice where sacrificed at 2.5 days post coitus and the embryos were collected by oviduct flushing. The isolation of ES cells was performed in the presence of two inhibitors (2i): PD0325901 (1μM) and CHIR99021 (3μM). 2i was always used during experiments at the ES cell stage. Neural differentiation was performed according to already published protocols with minor changes (Bibel et al., 2004, 2007). ES cells derived in 2i medium (N2B27 medium plus 2i and LIF) were adapted for some passage in standard ES medium (DMEM, 15% ES screened FBS, 2mM L-glutamine 0.1 mM non-essential amino acids, 0.1mM 2-mercaptoethanol and LIF) always plus 2i. The neural differentiation was then performed as previously described (Bibel et al., 2004) and 2i was removed from media at the moment of cells suspension for cellular aggregate formation. Cells were collected for analysis at 72h, 10D, and 15D after neural precursor plating. To activate Cre recombinase, 4-hydroxytamoxifen treatment was administrated in four pulses at 300nM concentration at the following time-points: 2h after neural precursors plating, and again 24 h, 48 h, and 5 days after plating.

In this way, we established and proficiently differentiated into glutamatergic neurons two *Ezh2*^flox/flox^*/CreERT2^/+^/eYFP^/+^* mESCs lines, referred to as M3 and M6. We also obtained one *Ezh2*^WT/WT^*/CreERT2^/+^/eYFP^/+^*mESCs line, for which, however, we did not achieve proper neuronal differentiation.

### qPCR

RNA was extracted using Qiagen RNeasy^®^ Plus Mini kit according to manufacturer instructions. cDNA was synthesized using SuperScript^®^ VILO™ cDNA synthesis kit according to manufacturer instructions. qRT-PCR was performed on 7900HT Fast Real-Time PCR system (Applied Biosystem). qPCR primers are listed in Supplementary Table 11. qPCR data were analyzed as previously described (Livak and Schmittgen, 2001) and data are expressed as fold change compared to the first time point of the time course (CTR 72h and 4-OHT 72h).

### Protein Extraction and Immunoblotting

Cell lysate was obtained using RIPA buffer (10mM Tris-Cl pH 8.0, 1% Triton, 0,1% SDS, 0,1% Deoxycholate, 140mM NaCl, 1mM EDTA). Western Blot was performed according to standard procedure using NuPage 4-12% bis-tris gel precast (Invitrogen). If reprobing of the same membrane was performed, stripping was carried out by incubation with stripping buffer (0.1M glycine, 20 mM magnesium acetate, 50 mM KCl, ph 2.2) for 10’, 2 washes with stripping buffer, 3 washes with TBST buffer and re-blocking. Western blot was performed with the following antibodies: mouse anti-EZH2 1:100 (in-house made), anti-H3K27me3 1:1000 (#9733 Cell Signalling), anti-GluR1:1000 (#04-855 Milllipore), anti-synaptophysin 1:500 (SAB4502906 Sigma), anti-β-actin 1:1000 (A4700 Sigma), anti-H3 1:2500 (#06-755 Millipore). After signal detection, X-ray films were scanned and images were processed only by cropping. Densitometric analysis was performed using ImageJ.

### Statistical Analyses

Densitometric data from Western blot signal were normalized against housekeeper genes (β-actin or vinculin for EZH2 and H3 total for H3K27me3). The fold change of 4OHT and CTR samples was calculated for each time point. One sample t-test was applied for every time point setting at 1 the value of controls. Unpaired t-test was applied to qPCR data expressed as fold change compared to the first time point of the time course (CTR72h and 4-OHT 72h).

### RNA-seq and ChIP-seq

RNA was extracted using Qiagen RNeasy^®^ Plus Mini kit according to manufacturer instructions. cDNA library preparation was performed according with Illumina protocol using poly-A enrichment. Sequencing was performed on Illumina HiSeq 2K using 100bp paired end reads. For ChIP-seq cells were cross-linked in 1% formaldehyde for 10 min and the cross-liking was stopped by incubating in 0.125 M glycine for 5 min. After washing cells were harvested in SDS buffer (0.5% SDS, 50mM Tris-Cl pH 8.1, 100mM NaCl, 5mM EDTA pH 8, 0.02% NaN_3_). Prior to sonication, the lysate was diluted 2:1 with dilution buffer (5% Triton X-100, 100mM Tris-Cl pH 8.6, 100mM NaCl, 5mM EDTA pH 8.5, 0.02% NaN_3_). Sonication was performed with Covaris sonicator generating DNA fragments with a bulk size around 250bp. Chromatin was quantified with Lowry method. After sonication, 600μg of chromatin and 12μg of anti-H3K27me3 (#9733BF Cell Signalling) were used for immunoprecipitation overnight at 4°C and recovered the following day using Dynabeads Protein G (Life Technologies). 1% of chromatin prior to immunoprecipitation was used as input. Immunoprecipitate was washed three times in low salt and one time in high salt wash buffer (1% Triton-X, 150mM or 500mM NaCl, 20mM Tris-Cl pH 8.0, 0.1% SDS, 2mM EDTA pH 8.0). One additional wash was performed in TE. Elution and crosslink reverse was performed under standard condition. DNA was then purified using PCR purification kit (QIAGEN). Samples for ChIP-seq were prepared according to Illumina ChIP-seq sample preparation kit and DNA was sequenced on Illumina HiSeq 2000 platform.

### Bioinformatic Analysis

Statistical analysis was performed using R 3.1.2 statistical software^1^.

Correlation matrix plot has been generated using ‘corrplot’ package^2^.

Heat maps were generated using ‘‘pheatmap’’ package^3^.

Enrichment analyses for canonical pathways were performed using IPA software (IPA, QIAGEN Redwood City^4^.

Gene set enrichment analysis was performed using GSEA software (Mootha et al., 2003; Subramanian et al., 2005) from http://www.broadinstitute.org/gsea/index.jsp

Matrix for ChIP-seq heat map was generated using HOMER (Heinz et al., 2010). Clustering and visualization was performed with Cluster 3.0 (de Hoon et al., 2004) and Java Treeview (Saldanha, 2004) respectively.

PRDM13 regulatory network was generated using GeneMania (Warde-Farley et al., 2010) https://genemania.org/.

### RNA-seq Analysis

Reads were aligned to mouse reference genome (mm9) using TopHat1.4 (Trapnell et al., 2012). Quantification was performed at gene level using featureCount (Liao et al., 2014). Raw counts were used as input for Edger, which has been used to produce normalized gene expression values (count per million or ‘cpm’) and to perform differential expression analysis.

### ChIP-seq Analysis

Reads were aligned to the genome (mm9) using Bowtie v.0.12.9 allowing up to two mismatches per read. Enriched regions for H3K27me3 were identified using MACS2 version 2.1 (Zhang et al., 2008) for broad peak calling with parameters q-value 0.1, broad-cut-off 0.1 and –nomodel. H3K27me3 target genes were identified as genes with an enriched region in the span of ± 2.5kb around their transcription start site.

## Data Availability Statement

The original contributions presented in the study are publicly available. This data can be found here: https://www.ebi.ac.uk/arrayexpress/experiments/E-MTAB-11363/ and https://www.ebi.ac.uk/arrayexpress/experiments/E-MTAB-11387/.

## Ethics Statement

Ethical review and approval were not required for the study on human participants in accordance with the local legislation and institutional requirements. The Italian legislation, at the time of approval of these experiments, did not require a specific ethical review process for all the experiments involving animals. A central (Government) review was required only for particular species (e.g., dogs, cats, and non-human primates) or for experiments done without anesthesia or that will or may cause severe pain. In all other cases, only a notification of the experiments to the Ministry of Health was required. Accordingly, the experiments involving animals included in this paper have been notified to the Italian Ministry of Health in 2009, before ARRIVE guidelines implementation. Experiments involving animals have been performed in accordance with the Italian Laws (D.L.vo. 116/92 and following additions), which enforced EU 86/609 Directive (Council Directive 86/609/EEC of 24 November 1986 on the approximation of laws, regulations, and administrative provisions of the Member States regarding the protection of animals used for experimental and other scientific purposes). Mice have been housed accordingly to the guidelines set out in Commission Recommendation 2007/526/EC - June 18, 2007 on guidelines for the accommodation and care of animals used for experimental and other scientific purposes.

## Author Contributions

SB performed the mice work, ESC isolation and lines establishment, neuronal differentiations, samples collection, stainings, western blots, RNAseq and ChIP-seq experiments and library preparations. CR shared her expertise about the neuronal differentiation system used in this work. SB, JH, and ST performed the qPCR analyses. PL, VD, and ST performed the bioinformatic analyses. JH helped with data analysis and interpretation. ST, JH, PL, and SB wrote the manuscript and prepared the figures with contributions from all authors. GT conceived, designed, and supervised the study.

## Conflict of Interest

PL is Director of Single-Cell Systems Biology at DarwinHealth, Inc., New York, NY, United States. VD currently works as a Post-Doctoral Researcher in Novo Nordisk Research Center Seattle, Inc. The remaining authors declare that the research was conducted in the absence of any commercial or financial relationships that could be construed as a potential conflict of interest.

## Publisher’s Note

All claims expressed in this article are solely those of the authors and do not necessarily represent those of their affiliated organizations, or those of the publisher, the editors and the reviewers. Any product that may be evaluated in this article, or claim that may be made by its manufacturer, is not guaranteed or endorsed by the publisher.

## Footnotes

1
https://www.r-project.org/

2
https://cran.r-project.org/web/packages/corrplot/index.html

3
https://cran.r-project.org/web/packages/pheatmap/index.html

4
http://www.qiagen.com/ingenuity
