# Supplementary material for: EZH2-Mediated H3K27me3 Targets Transcriptional Circuits of Neuronal Differentiation
Source: Front Neurosci. 2022 May 12;16:814144. doi: 10.3389/fnins.2022.814144 (PMC9133892; doi:10.3389/fnins.2022.814144)
Supplement: Supplementary file 1 [file Data_Sheet_1.zip › 7_SupplementaryFullLengthBlots.pdf]

Full length gel Fig1B

Acquisition A

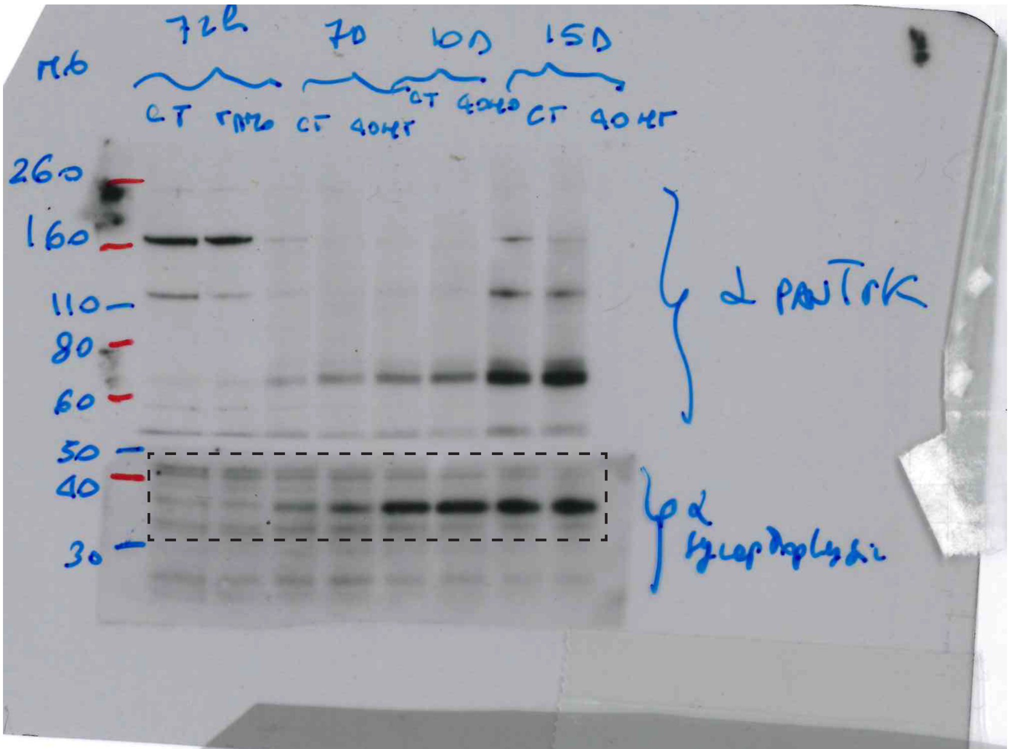

Pan-TRK detection.
(Not showed in this paper)

Synapthophysin detection

Acquisition B

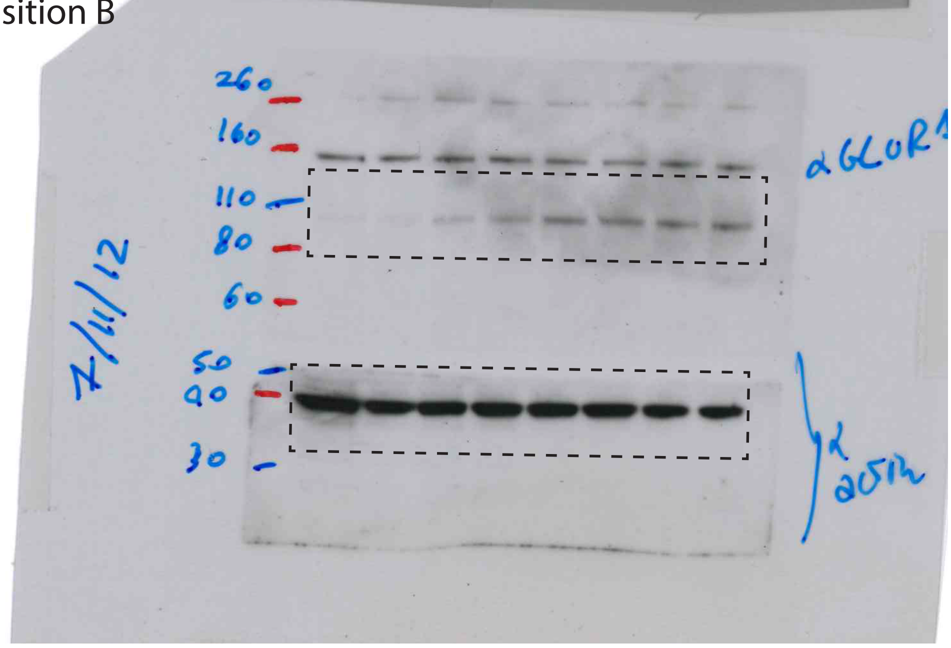

GluR1 detection

β-actin detection

Full length gel Fig1C and 1D - M3 line

Acquisition A

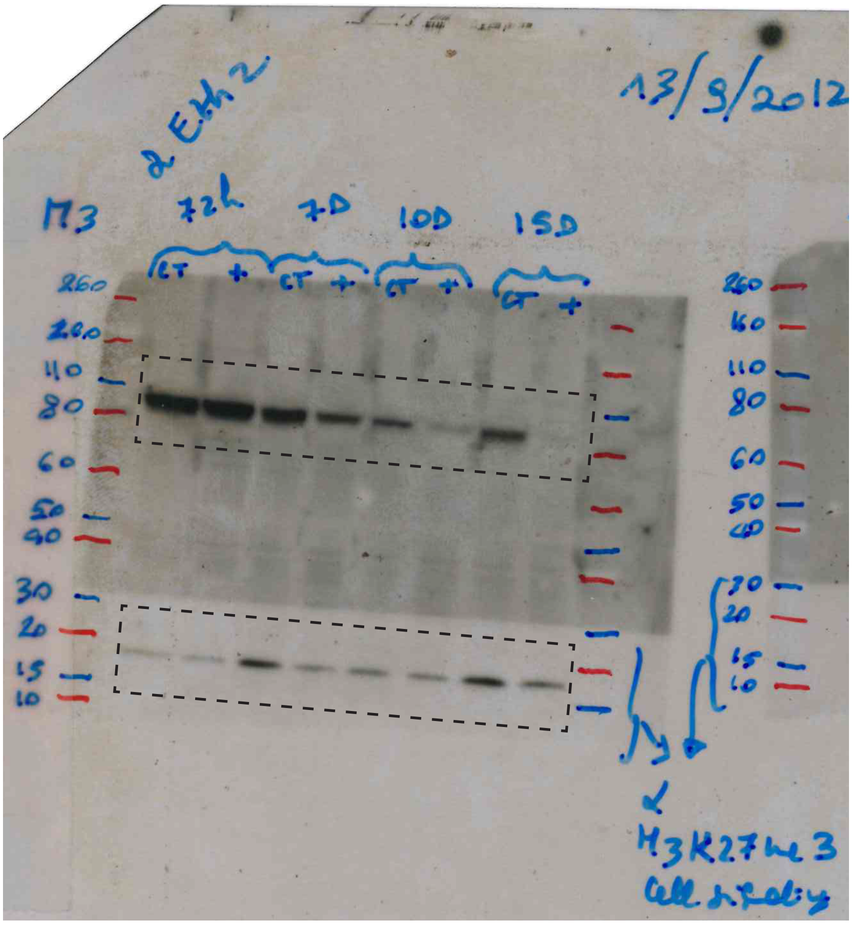

Ezh2 detection

H3K27me3 detection

Acquisition B

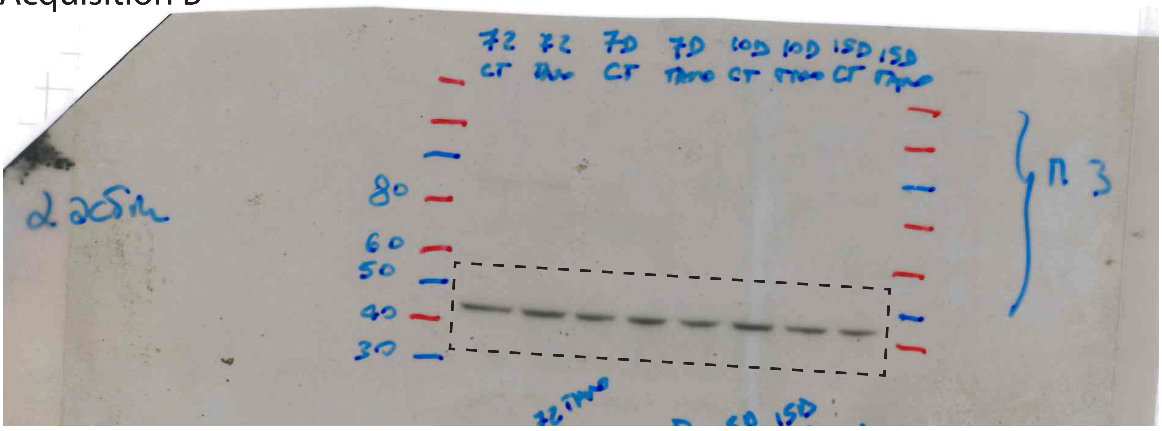

β-actin detection

Acquisition C

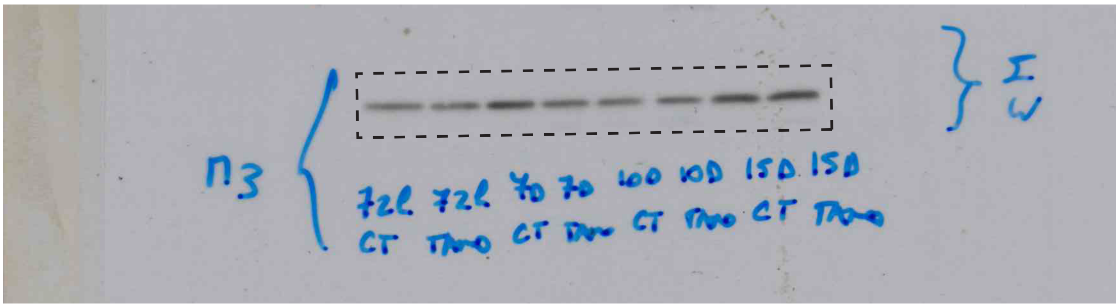

Histone H3 total detection

Full length gel Fig1C and 1D - M6 line

Acquisition A

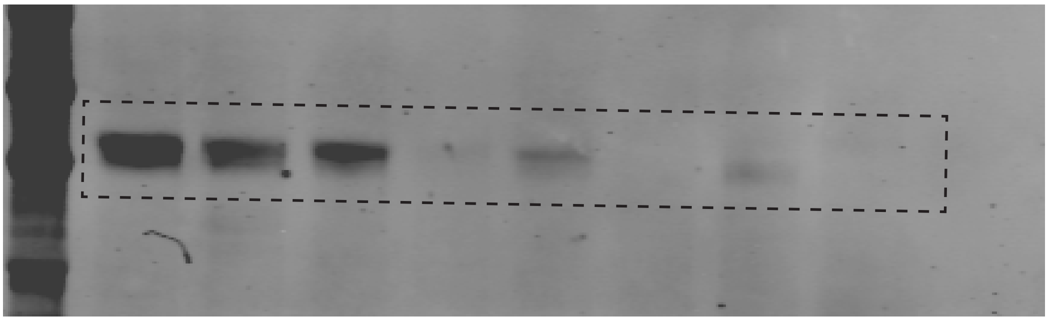

Ezh2 detection

Acquisition B

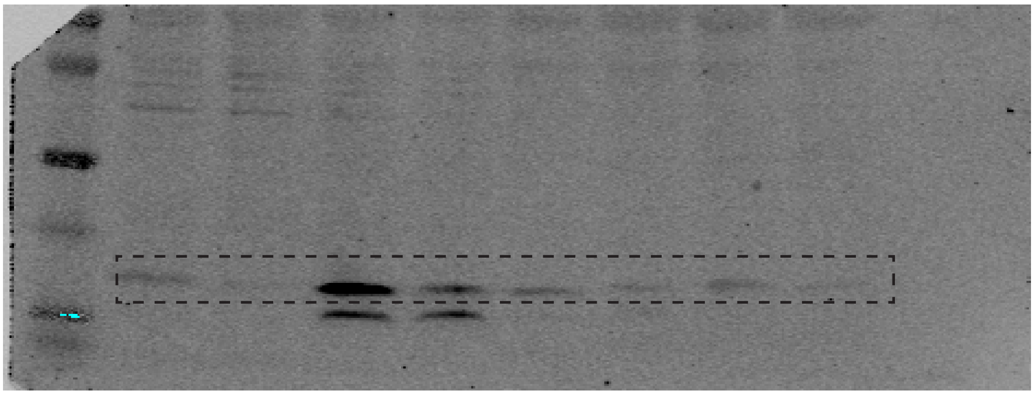

H3K27me3 detection

Acquisition C

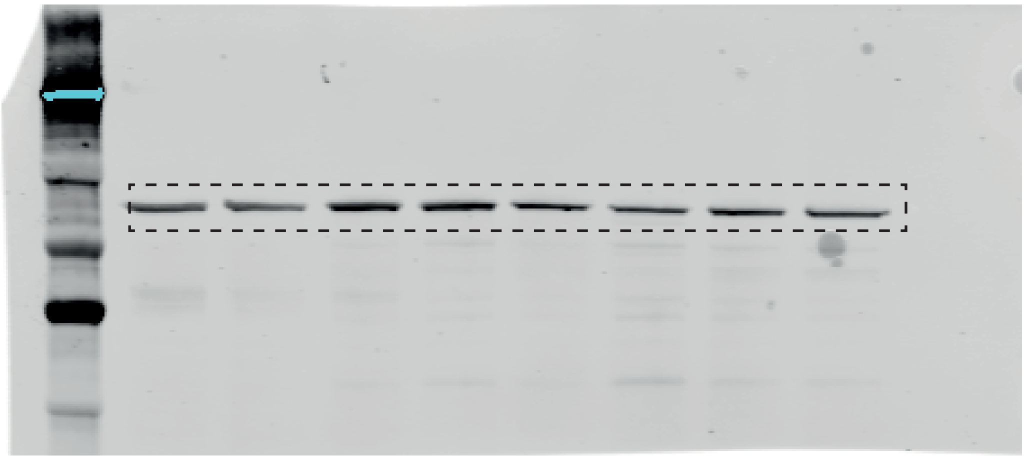

Vinculin detection

Acquisition D

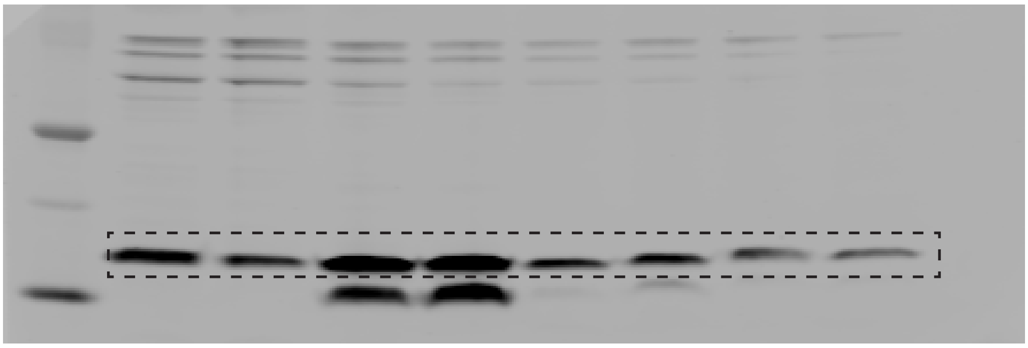

H3tot detection
