# Supplementary material for: EZH2-Mediated H3K27me3 Targets Transcriptional Circuits of Neuronal Differentiation
Source: Front Neurosci. 2022 May 12;16:814144. doi: 10.3389/fnins.2022.814144 (PMC9133892; doi:10.3389/fnins.2022.814144)
Supplement: Supplementary file 1 [file Data_Sheet_1.zip › 1_SupplementaryInformation.pdf]

Supplementary information

Supplementary figure legends

Figure S1. Ezh2flox/flox deletion and neuronal differentiation correctly occur upon 4-OHT treatment (A) Copy number assay for Cdnp are shown. Cdnp gene is located on chromosome 6 between ROSA26 and Ezh2 locus. DNA was extracted from control (+/+) and Ezh2 floxed lines (fl/fl) treated with 500nM 4-OHT for 72hrs. (B) Western blot analysis of EYFP expression during differentiation shows successful recombination. (C) Ezh2 mRNA levels measured by qRT-PCR verifies ablation after 48 hours of treatment with 4-OHT. (D) Representative images of differentiating neurons up to 72h after neuronal progenitors plating stained for Pax6 and Ki67.

Figure S2. Complete list of pathways H3K27me3 ChIP-seq. Heat map showing the complete list of significantly enriched canonical pathways (p-value<0.001) showed in figure 2d. P-values were computed using IPA software.

Figure S3. Heatmap of top variants genes. Heatmap showing the unsupervised hierarchical clustering of the top variant genes in 4-OHT and CTR samples.

Figure S4. Gene Set Enrichment Analysis for glutamatergic genes. GSEA plots for glutamatergic signature at 10D and 15D.

Figure S5. (A) and (B) Canonical pathway analysis performed on the downregulated DEGs (FDR < 0.05) at 10D and 15D, respectively, found to be H3K27me3 targets in CTR10D. The top 15 pathways are shown. B-H multiple testing corrected p-values were computed using IPA software. (C) and (D) Canonical pathway analysis performed on down-regulated DEGs (FDR < 0.05) at 10D and 15D, respectively. The top 15 pathways are shown. B-H multiple testing corrected p-values were computed using IPA software.

Supplementary table legends

Supplementary Table 1. List of H3K27me3 targets specific for CTR10D samples that fail H3K27me3 acquisition in 4-OHT samples.

Supplementary Table 2. List of differentially expressed genes with FDR < 0.05 between CTR and 4-OHT samples at 72h, 10D and 15D.

Supplementary Table 3. Canonical pathway analysis of upregulated DEGs in 4OHT10D samples that are H3K27me3 targets in CTR10D samples.

Supplementary Table 4. Canonical pathway analysis of upregulated DEGs in 4OHT15D samples that are H3K27me3 targets in CTR10D samples.

Supplementary Table 5. Canonical pathway analysis of downregulated DEGs in 4OHT10D samples that are H3K27me3 targets in CTR10D samples.

Supplementary Table 6. Canonical pathway analysis of downregulated DEGs in 4OHT15D samples that are H3K27me3 targets in CTR10D samples.

Supplementary Table 7. Canonical pathway analysis of upregulated DEGs in 4OHT10D samples with FDR < 0.05.

Supplementary Table 8. Canonical pathway analysis of upregulated DEGs in 4OHT15D samples with FDR < 0.05.

Supplementary Table 9. Canonical pathway analysis of downregulated DEGs in 4OHT10D samples with FDR < 0.05.

Supplementary Table 10. Canonical pathway analysis of downregulated DEGs in 4OHT15D samples with FDR < 0.05.

Supplementary Table 11. List of primers used in this work.
