# Supplementary figures and images for: EZH2-Mediated H3K27me3 Targets Transcriptional Circuits of Neuronal Differentiation
Source: Front Neurosci. 2022 May 12;16:814144. doi: 10.3389/fnins.2022.814144 (PMC9133892; doi:10.3389/fnins.2022.814144)

Figure S3

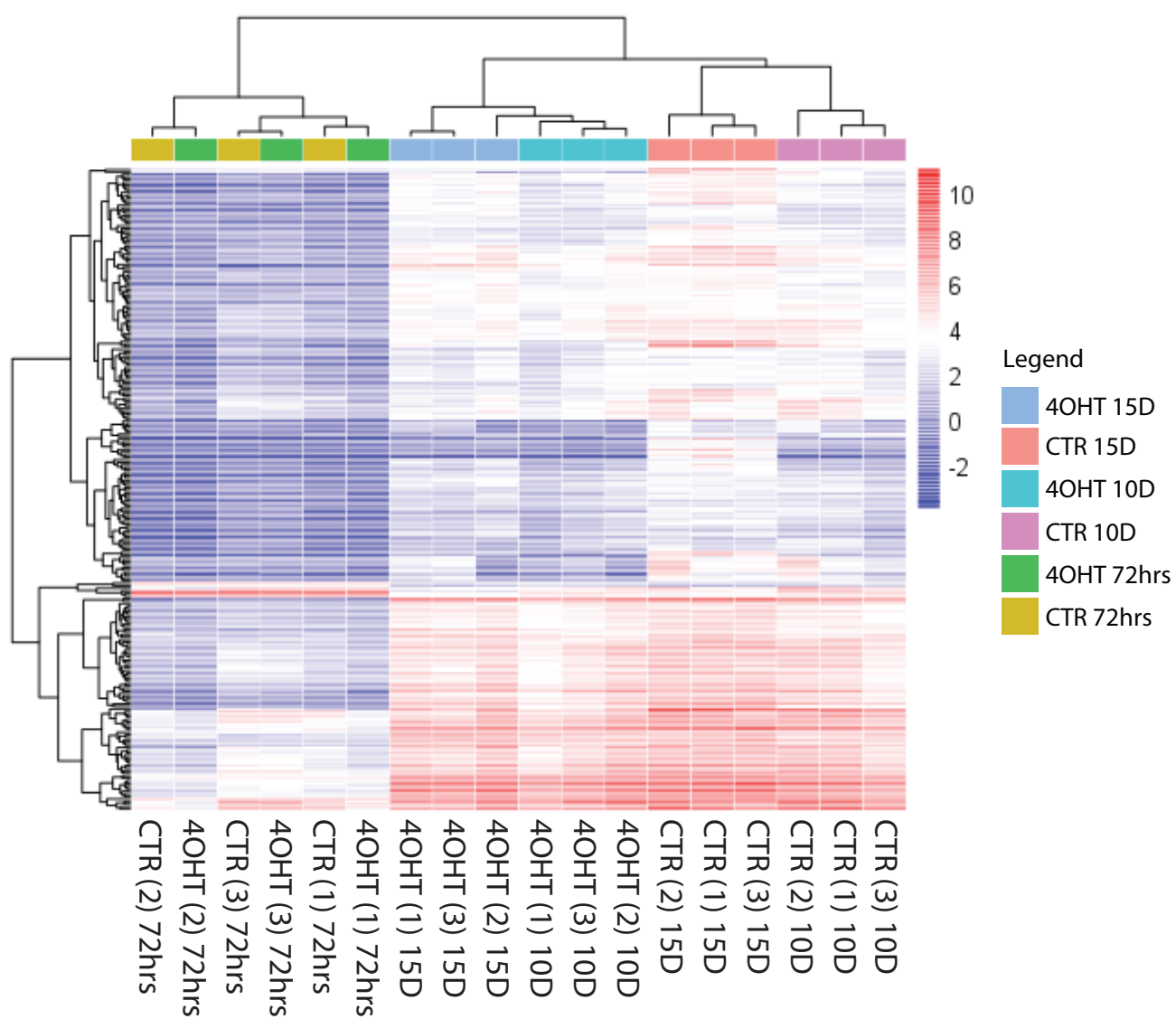

Supplement: Supplementary file 1 [file Data_Sheet_1.zip › 4_Figure S3.pdf]

## Gene Set Enrichment Analysis for glutamatergic genes

10 Days

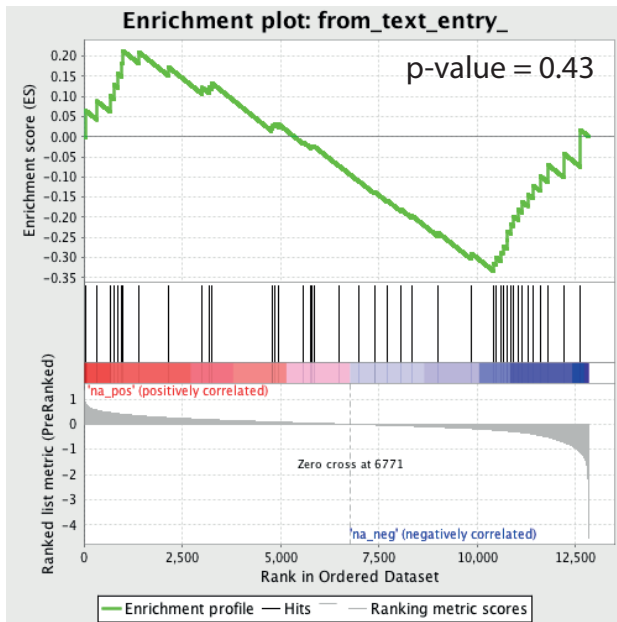

15 Days

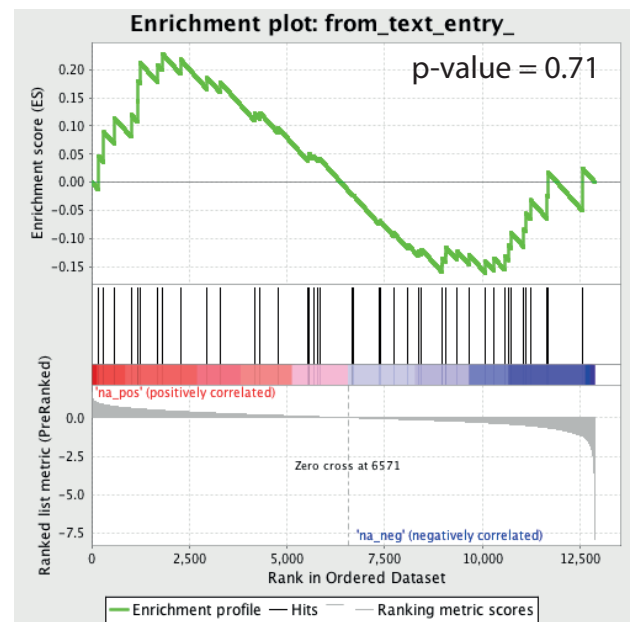

Supplement: Supplementary file 1 [file Data_Sheet_1.zip › 5_Figure S4.pdf]

Figure S5

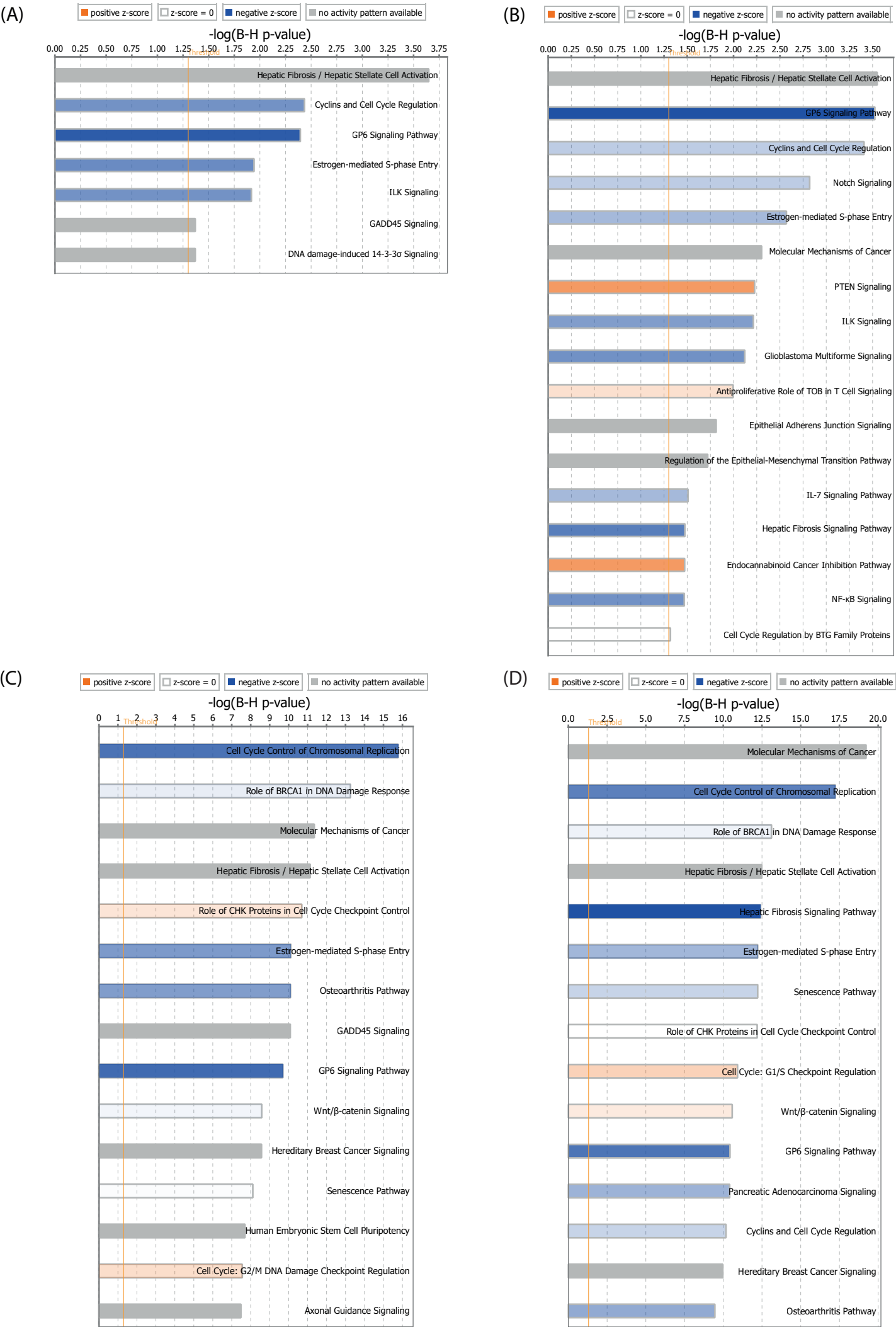

Supplement: Supplementary file 1 [file Data_Sheet_1.zip › 6_Figure S5.pdf]

Figure S1

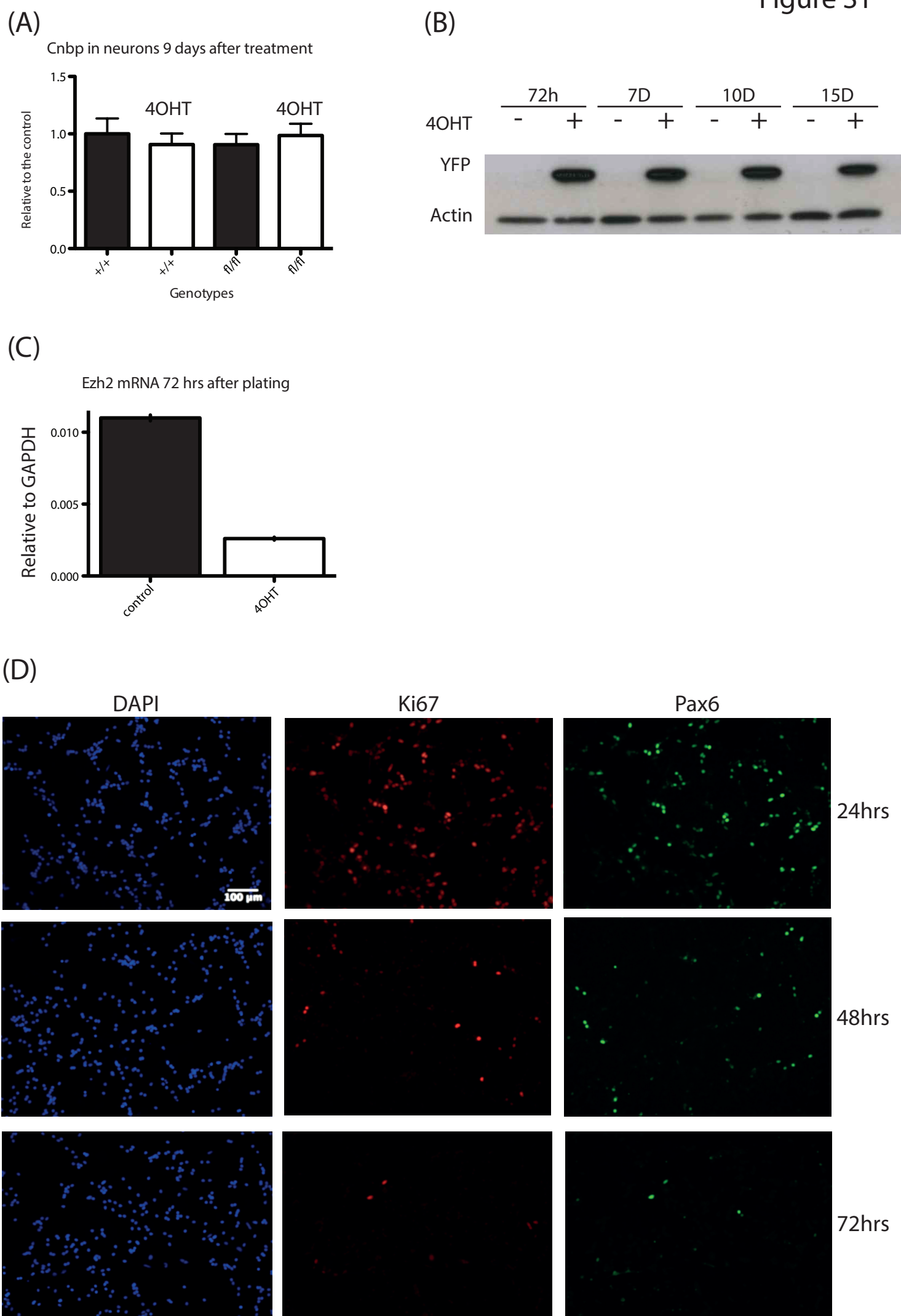

Supplement: Supplementary file 1 [file Data_Sheet_1.zip › 2_Figure S1.pdf]

Figure S2

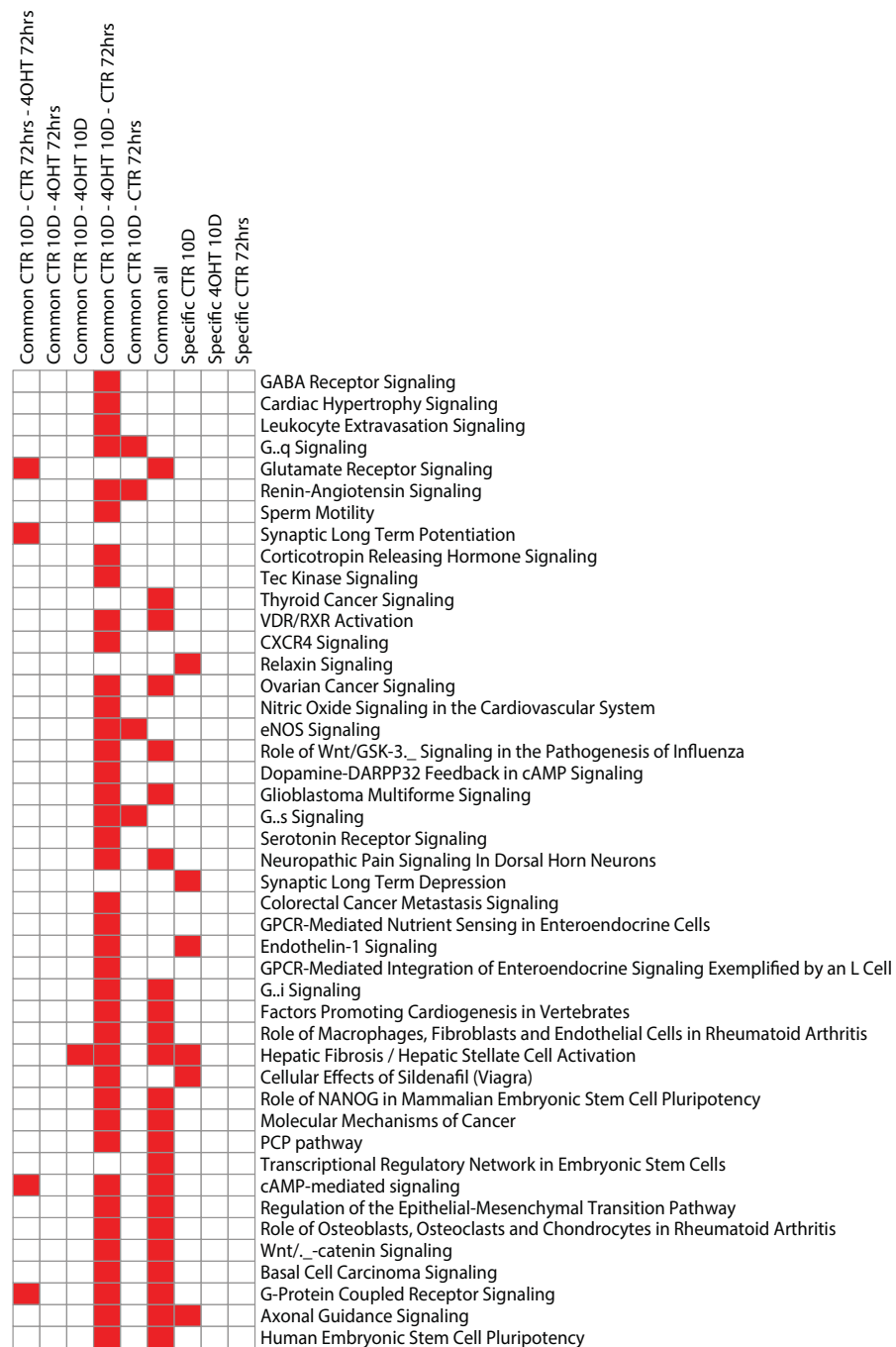

Supplement: Supplementary file 1 [file Data_Sheet_1.zip › 3_Figure S2.pdf]
